# Phylogenetic characteristics and recombination analysis of echovirus 5 associated with severe acute respiratory infection in China

**DOI:** 10.1128/spectrum.01711-23

**Published:** 2023-10-11

**Authors:** Wenxia Li, Jinhua Song, Jin Xu, Huiling Wang, Hongjian Duan, Yong Zhang, Wenbo Xu, Hua Fan, Yan Zhang

**Affiliations:** 1 National Health Commission (NHC) Key Laboratory of Medical Virology and Viral Diseases, National Institute for Viral Disease Control and Prevention, Chinese Center for Disease Control and Prevention, Beijing, China; 2 School of Public Health, Shandong First Medical University & Shandong Academy of Medical Sciences, Jinan, China; 3 Institute of Expanded Immunization Programme, Henan Provincial Center for Disease Control and Prevention, Zhengzhou, China; Oklahoma State University College of Veterinary Medicine, Stillwater, Oklahoma, USA

**Keywords:** echovirus 5, severe acute respiratory infection, phylogenetic characteristic, molecular epidemiology, recombination

## Abstract

Echovirus 5 (E5) belongs to the enterovirus B (EV-B) species, which is one of the pathogens causing respiratory diseases. Only two whole genome sequences (WGSs) of E5 are available in GenBank, and few studies on E5 have been reported worldwide. In this study, human rhabdomyosarcoma (RD) cells were used to isolate E5 from respiratory samples associated with severe acute respiratory infection (SARI). The nucleotide sequence of the VP1 region and the WGSs of two E5 strains were sequenced using Sanger sequencing and high-throughput sequencing technology, respectively. Together with 31 E5 VP1 sequences and 2 WGSs downloaded from GenBank, a total of 33 complete VP1 sequences and 4 WGSs were used for genetic characterization and evolutionary analysis. Phylogenetic analysis revealed five genotypes (A–E) based on complete VP1 sequences. Evolutionary analysis showed that the time to the most recent common ancestor of E5 was approximately 1952 (95% highest posterior density [HPD] range, 1948–1953]), with a mean substitution rate of 7.74 × 10^−3^ subs/site/year (95% HPD range, 6.44–9.11 × 10^−3^). In addition, the WGS analyses showed that the nucleotide and amino acid similarities of the coding regions of the two strains obtained in this study with the prototype of the E5 strain Noyce were 80.2% and 97.2%, respectively. Recombination analysis showed that the two E5 strains may have intraspecific recombination with E6, EV-B85, and CVA9 serotypes in the nonstructural region. This study reports the isolation of E5 from SARI cases for the first time, and genetic characterization and evolutionary analysis provide valuable information on global E5 molecular epidemiology.

**IMPORTANCE:**

This study is the first report of echovirus 5 (E5) associated with severe acute respiratory infection and obtained the first E5 whole-genome sequence in China. Combined with the sequences available in the GenBank database, the first genotyping, phylogenetic characteristics, recombination, and genetic evolutionary analysis of E5 was performed in this study. Our findings providing valuable information on global E5 molecular epidemiology.

## INTRODUCTION

Human enteroviruses (EVs) are RNA viruses belonging to the genus *Enterovirus* and the family *Picornaviridae*. The International Committee on Taxonomy of Viruses (ICTV) classified EVs into four species (enterovirus A–D) based on nucleotide (nt) variability in the VP1 region (1). Echovirus 5 (E5) belongs to the *Enterovirus B* (EV-B) species. The genome is approximately 7.5 kb and contains a long open reading frame (ORF) ﬂanked by a 5′ untranslated region (UTR) and a 3′UTR. The ORF is 6,588 nt and can be translated into 2,196 amino acids, which can be cleaved into three polyprotein precursors, P1, P2, and P3. P1 encodes four structural proteins, VP1–VP4, while P2 and P3 encode nonstructural proteins 2A–2C and 3A–3D, respectively (2). The 5′UTR contains multiple stem‒loop structures associated with viral replication and translation, and the 3′UTR has a vital role in viral replication (3, 4). In 1999, Oberste et al. found that the VP1 coding region carries major neutralization epitopes among the capsid proteins and is likely to be the best region for virus identification and molecular typing (1). The molecular typing method based on VP1 coding region variation is now used to confirm serotypes. The genotype classification analysis of most enteroviruses, such as EV-71, CVA9, CVA8, CVA6, and E5 (5–9), is based on this approach. Enteroviruses have been isolated from cases of various diseases, including aseptic meningitis, meningoencephalitis, and gastrointestinal and respiratory diseases (10–13). The prototype strain of Noyce was isolated in the United States in 1954 from patients with aseptic meningitis (14). Subsequently, aseptic meningitis outbreaks due to E5 were reported in Finland and Korea (15, 16). However, few studies on E5 associated with respiratory infections have been reported until now. To date, only two studies of E5 whole genome sequences (WGSs) have been published (17, 18), and the WGS of E5 circulating in China has not been reported. In this study, we first performed whole-genome sequencing of E5 strains circulating in China in 2018/2019 using high-throughput sequencing (HTS) technology. The genotype distribution and the WGS genetic characteristics were analyzed in this study. Our study of E5 strains from respiratory samples provided essential scientific data for studying the molecular epidemiological and genotype distribution of E5.

## RESULTS

Four hundred pharyngeal swab samples from SARI patients in Luohe Central Hospital, Henan Province from October 2017 to May 2021 were tested, and two E5-positive samples were detected, of which one sample (SA18-334/E5i/Luohe-henan-China/2018) was successfully isolated and the other sample (SA19-378/E5s/Luohe-henan-China/2019) failed.

### VP1 phylogenetic analysis and genotyping of E5

**Fig 1:**
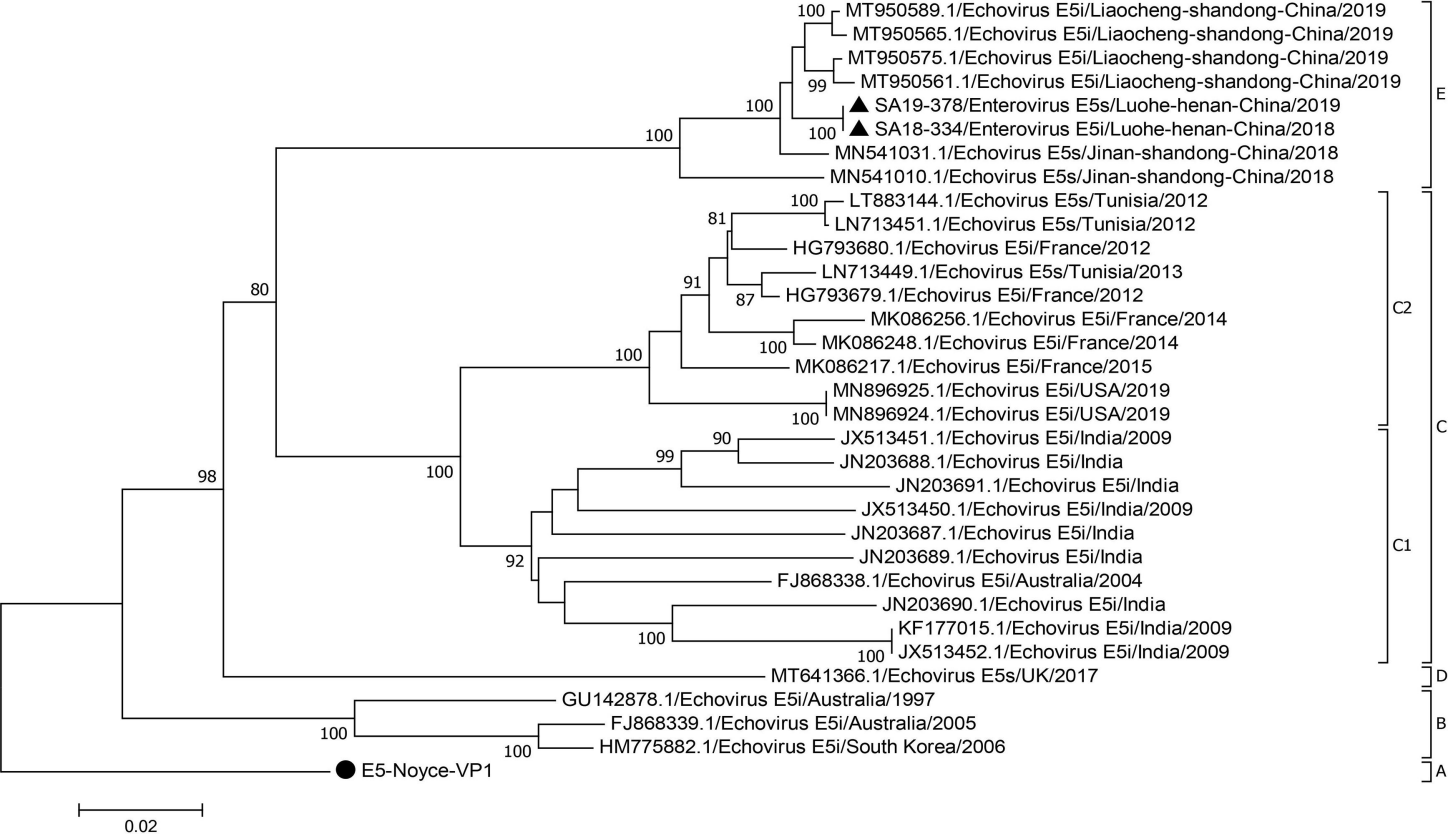
Maximum-likelihood phylogenetic tree based on the complete VP1 coding region sequences of E5 strains available from GenBank. The two E5 strains in this study are indicated by ▲. The prototype of the E5 strain is indicated by ●. The numbers at the nodes indicate the bootstrap support for the node (percentage of 1,000 bootstrap replicates).

The two E5 sequences obtained in this study together with the complete VP1 coding region of 31 global E5 sequences were used to construct a maximum likelihood (ML) phylogenetic tree. The results showed that the 33 sequences could be classified into five genotypes: A to E (Fig. 1). The mean distance between the five genotypes was 14.4%–20.2% and was larger than the intragenotype mean distances of 2.4%–9.6%, indicating the reliability of genotyping (Table 1). The prototype strain Noyce isolated in 1954 was named genotype A and the rest were named according to the time of isolation. Genotype B strains were isolated in Australia and South Korea. Genotype C strains can be further divided into subtypes, C1 and C2, with subtype C1 isolated from India and Australia and subtype C2 consisting of American, French, and African Tunisian strains. Genotype D strains were isolated in the United Kingdom in 2017. Notably, the Chinese isolates matched genotype E in 2018 and 2019.

**TABLE 1:** Average divergence between different genotypes of E5^*a*^

Genotype	A	B	C	D	E
A	−	3.4%–4.1%	3.8%–5.8%	4.5%	4.1%–5.1%
B	14.4%–15.0%	−	2.7%–5.5%	2.7%–3.1%	2.7%–4.5%
C	17.5%–20.7%	17.1%–20.3%	−	2.7%–4.5%	2.4%–5.5%
D	18.0%	17.1%–17.7%	17.8%–19.6%	−	4.1%–5.1%
E	17.8%–18.6%	17.7%–19.1%	17.1%–19.9%	19.1%–20.2%	−

^*a*^ The data below the diagonal indicate nucleotide diversity (underlined), and those above the diagonal indicate amino acids (not underlined).

### E5 amino acid variant analysis

**Fig 2:**
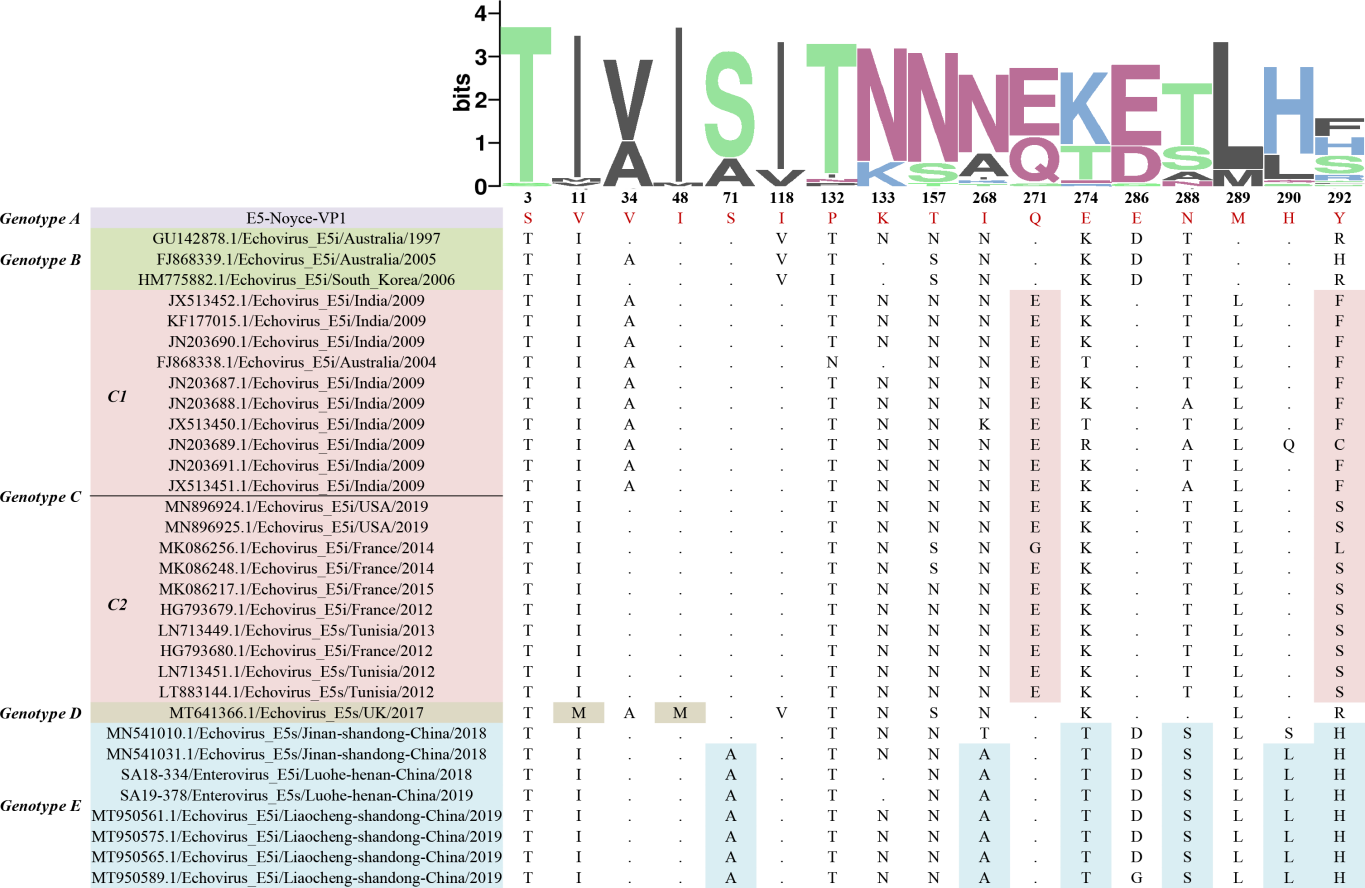
Polymorphic amino acid sites in the E5 VP1 region. WebLogo 3 was used for amino acid site presentation. Each logo consists of stacks of symbols, with one stack for each position in the sequence. The height of the symbols within the stack indicates the relative frequency of each amino or nucleic acid at that position. Red letters represent amino acids of the prototype strain (genotype A). Amino acids with pink, light brown, and blue backgrounds indicate genotypes C–E specific mutations, respectively.

Analysis of nucleotide polymorphisms in the complete VP1 region of 33 strains revealed that E5 had a nucleotide polymorphism Pi(Π) of 0.14, with a total of 373 mutation sites, mainly synonymous mutations (350). Meanwhile, 57 polymorphic sites were found, and S3T was the common amino acid mutation site compared to the prototype strain. Most E5 VP1 sequences belonging to genotypes C–E displayed specific amino acid substitutions. Genotype C sequences predominantly displayed amino acid changes at Q271E and Y292F/S. Genotype D contained two specific mutations, V11M and I48M, whereas genotype E contained six specific mutations, namely S71A, I268A, E274T, N288S, H290L, and Y292H (Fig. 2).

### Phylodynamic analysis

**Fig 3:**
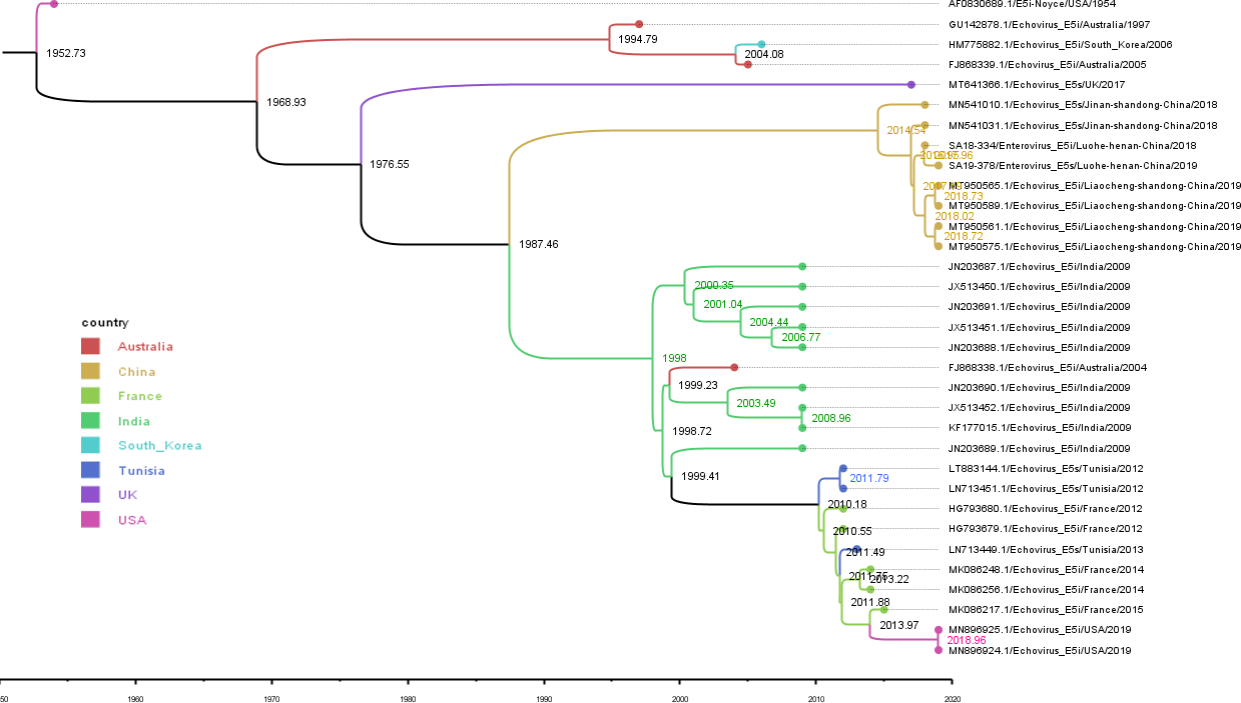
The MCC phylogenetic tree was generated using the MCMC method based on the complete VP1 sequences of 33 E5 strains and colored according to different countries. The *x*-axis is the time scale (years).

The maximum clade credibility (MCC) trees based on the 33 complete E5 VP1 sequences were generated using the Markov chain Monte Carlo (MCMC) method (Fig. 3). The evolutionary substitution rate for the complete VP1 region of E5 was 7.74 × 10^−3^ subs/site/year (95% highest posterior density [HPD] range, 6.44–9.11 × 10^−3^), with a predicted time to most recent common ancestor (tMRCA) of 1952 (95% HPD range, 1948–1953). Genotype D was suggested to have diverged in approximately 1977 (95% HPD, 1969–1972), and the tMRCAs of genotypes B, C, and E could be traced back to 1969 (95% HPD, 1960–1976), 1998 (95% HPD, 1996–2000), and 2014 (95% HPD 2013–2016), respectively.

### Whole-genomic characterization of the two E5 strains

Analysis using CLC Genomics Workbench 22.0 showed that the Q20 and Q30 values after trimming were 99.99%–100% and 96.47%–96.93%, respectively, and the N50 *de novo* assembly results were 1118 bp and 877 bp, respectively. Using the contig splicing results as reference sequences, the numbers of reads mapped to the reference sequences were 18,057,493 and 4,313,464, respectively. Almost the complete genome (7,446 nt/7,434 nt) could be assembled by *de novo* with an average sequencing depth of 140,369× and 100% coverage (Fig. S1), containing the complete coding sequence for the polyprotein and the 5′UTR (754 nt/742 nt) and 3′UTR (104 nt). The ORF of the two strains is 6,588 nt, which encodes a polypeptide of 2,196 amino acids. The base compositions of the two strains are 27.9%–28.0% A, 23.5% C, 24.5%–24.6% G, and 24% T. The WGS nucleotide and amino acid similarities between the two strains were 99.9% and 100.0%, respectively. The WGSs showed 80.5%–80.6% nucleotide identity and 97.2% amino acid identity with the E5 prototype, and one nucleotide deletion was found at position 104 and two nucleotide insertions at positions 573 and 7,342. Moreover, the proteins and genes with the highest sequence identity to the prototype strain were the P1 region (81.5%–81.6%) and VP4 region (84.5%), and the lowest sequence identity was observed for the P3 region (78.7%) and 3C region (79.2%), respectively. The two strains show higher similarity with E5 strains (MT950565 and MN541031) in the P1 and P2 regions; in the P3 regions, the two strains show greater identity with some EV strains, such as E6 strains (MF678307), suggesting that recombination occurs in these coding regions (Table S3).

### Recombination analysis of the henan E5 strains

**Fig 4:**
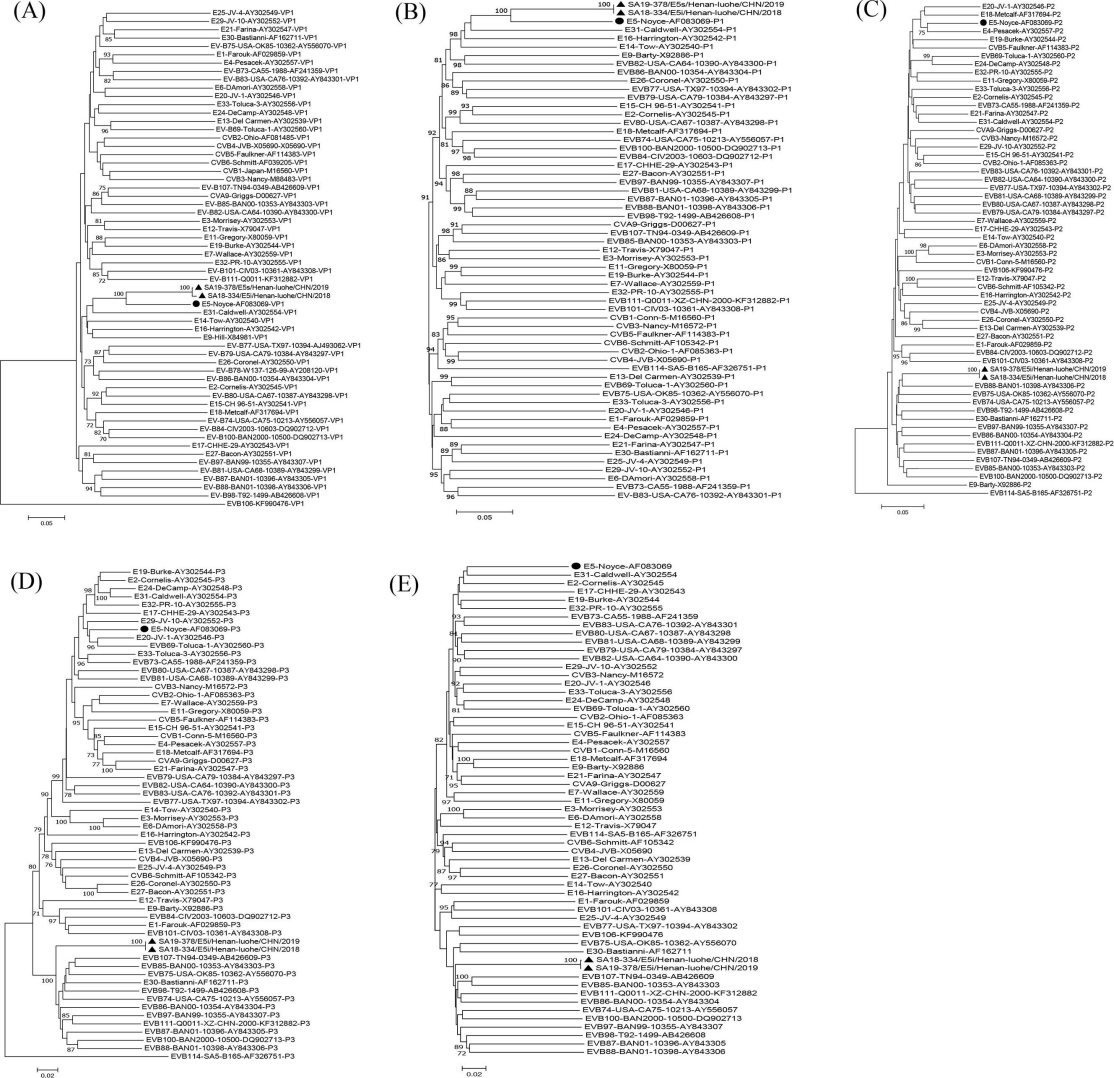
Neighbor-joining phylogenetic trees based on the VP1, P1, P2, and P3 coding regions and complete genome of EV-B and Henan E5 strains. Numbers at the nodes indicate bootstrap support for the node (percentage of 1,000 bootstrap replicates). (**A**) VP1 coding sequences. (**B**) P1 coding sequences. (**C**) P2 coding sequences. (**D**) P3 coding sequences. (**E**) Complete genome. The prototype of E5 is indicated by ●. The two E5 strains in this study are indicated by ▲.

To investigate the potential existence of recombination in E5, the prototype strains of EV-B (58 strains) were downloaded from GenBank together with the two E5 strains obtained in this study to construct the VP1, P1, P2, and P3 regions and the complete genome phylogenomic tree (Fig. 4). The phylogenetic trees based on the VP1 and P1 coding regions showed that E5 strains clustered with the prototype of E5, confirming the direct molecular typing results. Unlike the P1 phylogenetic trees, those of the P2 and P3 coding regions and the complete genome showed that the two strains clustered with the strains of other EV-B prototypes rather than with the E5 prototype, suggesting putative recombination in the genome of E5 strains.

**Fig 5:**
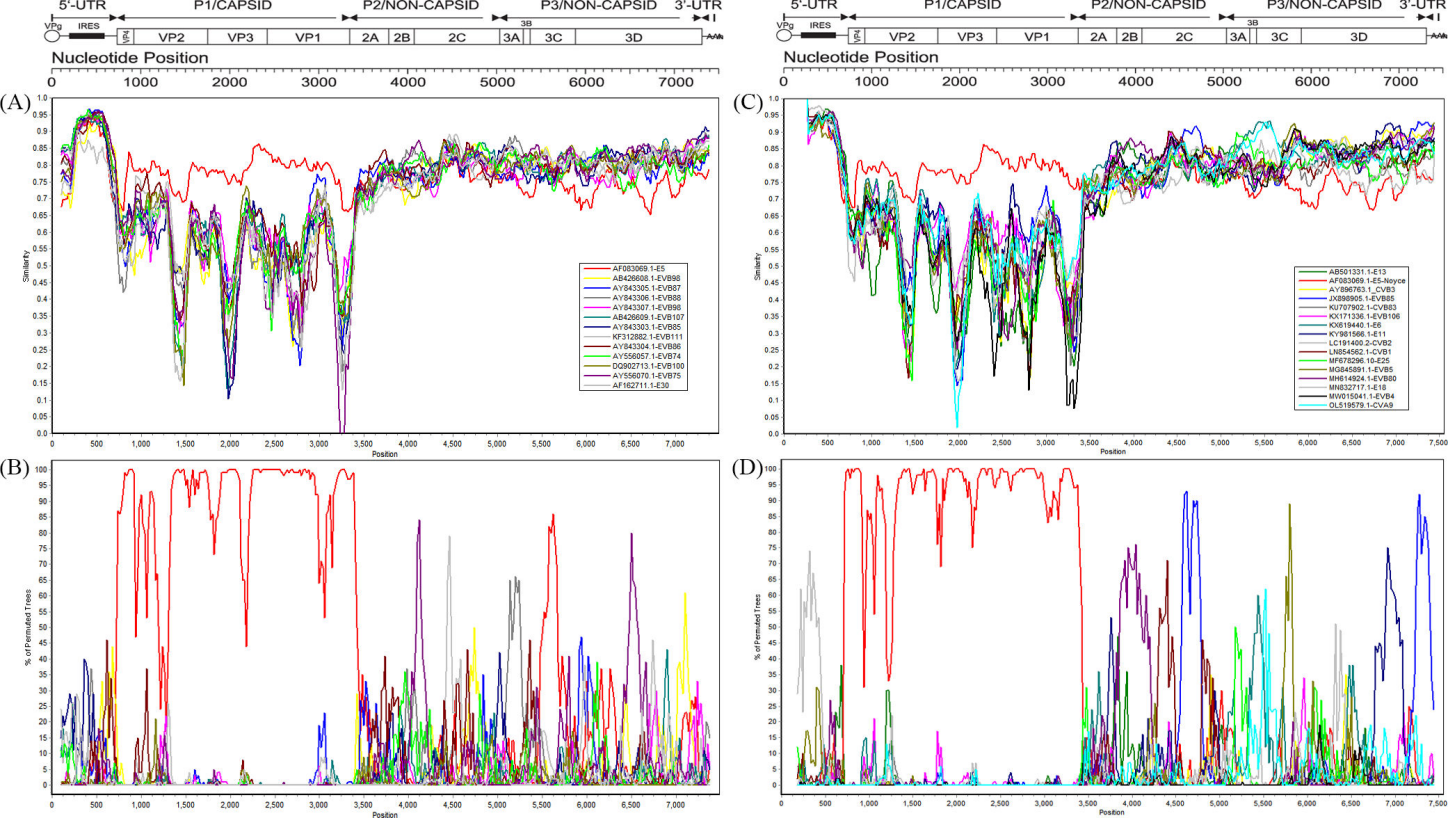
The two strains of Henan E5 were used as queries for recombination analysis by SimPlot. (**A** and **C**) Similarity plots. (**B** and **D**) Bootscanning analyses.

Based on the results of the phylogenetic analysis, the sequences that were more similar to E5 were selected for recombination analysis using SimPlot (Fig. 5A and B). The results showed that the Henan E5 strains were highly conserved in the P1 region. In the P2 and P3 regions, the two strains were highly similar to other EV-B prototypes, such as EV-B86 (nt position 3500–3900), EV-B75 (nt position 4000–4300), and EV-B88 (nt position bp 5100–5300). To further search for potential recombinant strains, we performed BLAST in GenBank with the P2 (2A–2C) and P3 (3A–3D) noncapsid regions of the Henan E5 strains. The results showed that the two E5 strains had high similarity with other serotypes in the nonstructural regions (Table S4). We screened the other non-E5 strains with complete sequences with >85% similarity to evaluated recombination events in the Henan E5 strains using similarity plots and bootscanning analysis (Fig. 5C and D). In the P1 region, the two E5 strains shared the highest similarity with the E5 prototype strain (AF083069.1). However, the two E5 strains shared the highest identity with the EV-B85 strain (JX898906/XJ-CHN/2011) in the 2C and 7,100 bp to 3′UTR regions. In the 3A–3B coding region, it shared the highest identity with the E6 strain (KX619440/Iran/2011) and CVA9 strain (OL519579.1/XZ-CHN/2018). The highest similarity with the E11 strain (KY981566/Israel/1999) was observed at the position of bp 6600–7100. This suggests that there may be small-scale recombination of Henan E5 strains in this region with the above serotypes.

## DISCUSSION

According to the U.S. National Enterovirus Surveillance System (NESS), from 1970 to 2005 E5 frequently observed in the 15 most common EVs, with a high rank of fourth in 1982. Children aged <1 year were the most common source for E5 detection, and CSF was the most common specimen. Fatal outcomes were reported in 3.8% of E5 infections with known outcomes (19). The epidemic of E5 has caused several outbreaks of aseptic meningitis, seriously endangering human health worldwide and causing a heavy disease burden (17). Information on E5 is less available in respiratory infection studies (13), which focus mainly on the gastrointestinal tract and central nervous system domains (10, 11, 20). Combining the complete VP1 region and WGS data available in GenBank, we performed the first whole-genomic characterization and evolutionary analysis of E5.

Accurate classification of genotypes helps discover the differences among genotypes and further understand the genetic evolution of viruses. We determined five genotypes in this study according to the genotype classification criteria of EVs. Currently, the prevalent E5 strains found in China are mainly genotype E. The E5 strains obtained in this study were clustered with isolates from Jinan and Liaocheng in Shandong Province, China, suggesting that the Henan E5 strains may have originated in Shandong Province. Given that the VP1 region can specifically neutralize antigens and affect neurotoxicity sites (5), we performed polymorphic site analysis and found 2, 2, and 6 specific mutant sites for genotypes C–E, respectively. The sites mentioned above may be necessary for delineating C–E genotypes. However, the role of amino acid mutations in the evolution of E5 strains needs to be verified by further virological studies, such as reverse genetics studies and animal experiments.

BEAST analysis revealed a global tMRCA for E5 dating back to 1952 and an average nucleotide substitution rate of 7.74 × 10^−3^ subs/site/year in the VP1 region, which was faster than the evolutionary rate of genotypes such as CVA9, CVB3, and EV-71 (21), suggesting the need for continuing enhanced monitoring of E5. Currently, the lack of global surveillance of E5 has resulted in the complete VP1 sequences in GenBank being very limited, thus making our results potentially biased. We collected as many complete VP1 region sequences of E5 as possible and excluded sequences that did not match the temporal signal provided by TempEst to ensure the accuracy and reliability of the results.

Previous studies have suggested that RNA genome recombination is one of the main drivers of RNA virus evolution, which is essential for the analysis of EV genetic evolution and to discover novel types (22, 23). The 3Dpol error-prone RNA-dependent RNA polymerases (RdRps) of EVs always lead to misincorporations during genome replication, resulting in a high mutation frequency in the replicating EV genome (22). Recombination affects virulence and the ability of the virus to spread in the nonstructural protein regions (P2 and P3) and may lead to outbreaks of this serotype (22, 24). EV-B is more prone to recombination events and has been reported for serotypes E7, CVA9, CVB3, and CVB5 (6, 25–27). The results of this study showed that the two E5 strains have high similarity with other serotypes in the nonstructural region, such as CVA9, E6, E11, and EV-B85, which were highly similar to the CVA9 and EV-B85 isolates in the Xinjiang and Tibetan regions of China in the 3C-3′UTR. It is hypothesized that Henan strains may be widely prevalent in China and undergo small-scale recombination with E6, CVB4, and CVA9 strains in the nonstructural region. Co-prevalence between virulent strains often leads to recombination events, which advance the continued evolution of the virus. Although fewer reports are available on E5, the multiple recombination events indicate that the E5 strains continue to evolve and are likely to become an important prevalent serotype of EVs in the future.

At present, HTS is a new technology that can compensate for the limitations of low viral load or the difficulty of virus isolation. In this study, we obtained the first E5 WGSs in China from respiratory samples using HTS technology and systematically analyzed the prevalence characteristics and genetic evolution of E5. It expands the number of E5 WGS in GenBank and enriches the relevant studies of E5 in SARI, providing baseline data for later studies related to E5. One study found that amantadine and ribavirin can be used to treat E5 infections (17). The significant genetic differences between the current prevalent and prototype strains suggest that the development and screening of antiviral drugs need to follow epidemiological changes and will continuously refine the prevalent strains. Meanwhile, the frequent occurrence of recombination events increases the difficulty of vaccine and drug development and poses a significant challenge to disease prevention and control. Therefore, more EV surveillance studies are needed to evaluate the widespread prevalence of nonpolio enteroviruses. And further enhance the association between EVs (such as E5) and EV-related diseases such as SARI. Meanwhile, full use of novel sequencing technologies is essential for a more accurate analysis of the genetic evolution and mutational variation patterns of E5 strains.

## MATERIALS AND METHODS

### Specimen collection

Pharyngeal swab samples from SARI patients were collected by the Henan CDC epidemiology staff and transported in sterile containers stored at 4°C–8°C to the Institute for Viral Disease Control and Prevention for further analysis.

### Virus isolation and molecular typing

According to the manufacturer’s instructions, viral RNA was directly extracted from clinical specimens using the Tianlong RNA Extraction Kit (Tianlong Biotechnology, Xian, China), and samples were screened using real-time RT‒PCR (28). All the above EV-positive samples were inoculated into human rhabdomyosarcoma cells for viral isolation. After complete EV-like CPE was observed, we harvested the infected cell cultures. The VP1 coding region sequences were amplified by RT‒PCR using the One Step RT‒PCR Kit (TaKaRa Biotechnology Dalian, China, cat: DRR057A) with the primers HEVBS1695 and HEVBR132 (29). The PCR products were identified by agarose gel electrophoresis with lengths of 1,000 nt. The nucleotide sequences were spliced upon comparison with the E5 prototype strains with Sequencher 5.0 (Gene Codes Corporation, Ann Arbor, MI, USA). The Enterovirus Genotyping Tool (https://www.rivm.nl/mpf/typingtool/enterovirus/) based on complete VP1 region sequences was used for EV serotyping.

### Next-generation sequencing

Viral RNA from clinical samples or viral isolates was extracted using the QIAamp Viral RNA Mini Kit (Qiagen, Hilden, Germany), and RNA quantification was performed using a Qubit 4 Fluorometer (Thermo Fisher Scientific, Waltham, MA, USA). Next-generation sequencing libraries were prepared using the VAHTS Universal V8 RNA-seq Library Prep Kit for MGI (Vazyme, China), and MiSeq sequencing using a 2 × 150 bp paired-end reads method was performed by Deep GenePlus-Shenzhen Clinical Laboratory. Clean reads from the viral database were *de novo* assembled into contigs using CLC Genomics Workbench 22.0 (QIAGEN, USA) with default parameters (30, 31). Contigs longer than 200 bp and with average coverage >30 were queried for further analysis.

### Bioinformatics analysis

Sequence alignment was conducted using MAFFT 7.475 (32). Additionally, the ML method or neighbor-joining method was used for phylogenetic tree construction with 1,000 bootstrap replicates in MEGA 7 (33), and mean nucleotide genetic distance (P-distance) and gene relatedness analyses were performed. The genotyping methodology was based on enterovirus A71 (EV-A71) genotypes, which were defined using a 15%–25% divergence threshold for the VP1 coding region. The best nucleotide substitution model (TN93+G+I) was selected by jModelTest (v2.1.7) (34). TempEst (v1.5.3) confirmed that the investigated sequences contained sufficient “temporal signal” for reliable estimation (35). The MCMC method implemented in BEAST (v1.10) was used to estimate the temporal phylogenies and rates of evolution (36). The 33 complete VP1-region sequences were analyzed using the uncorrected lognormal clock and constant site tree prior to the TN93+G+I nucleotide substitution model. A Bayesian MCMC run of 1 × 10^8^ generations was implemented with a sampling frequency of 1 × 10^4^ generations. The output from BEAST was analyzed using TRACER (v1.7.1). A MCC tree was generated using TreeAnnotator, and the results were subsequently visualized using FigTree (v1.4.3). Nucleotide and amino acid similarity analysis and polymorphism analysis were performed using BioEdit (v7.0.4.1) and DnaSP6, respectively (37). SimPlot was used to produce similarity plots with a 200 nt window moving in 20 nt steps to evaluate genetic diversity and detect recombination breakpoints (38).

### Sequence verification

To demonstrate the reliability of the *de novo* splicing results, the primers for E5 were designed by Oligo 7 to compare the *de novo* results (Table S1) (29, 39). The amplified regions overlapped between each primer pair to cover the whole genome. The 3ʹ end of the genome was amplified using the oligo-dT primer (7500 A) (40). The sequence results were obtained by comparison.

## Data Availability

The sequences generated in this study were submitted to GenBank with accession numbers OQ744532 and OQ744533.

## SUPPLEMENTAL MATERIAL

Supplemental material spectrum.01711-23-s0001.docx Tables S1-S4 and Figure S1.

ASM does not own the copyrights to Supplemental Material that may be linked to, or accessed through, an article. The authors have granted ASM a non-exclusive, world-wide license to publish the Supplemental Material files. Please contact the corresponding author directly for reuse.
