# Supplementary material for: Phylogenetic characteristics and recombination analysis of echovirus 5 associated with severe acute respiratory infection in China
Source: Microbiol Spectr. 2023 Oct 11;11(6):e01711-23. doi: 10.1128/spectrum.01711-23 (PMC10714939; doi:10.1128/spectrum.01711-23)
Supplement: Supplemental material — Tables S1-S4 and Figure S1. [file spectrum.01711-23-s0001.docx]

**SUPPLEMENTARY INFORMATION**

**TABLE S1** The primers designed in this study for amplifying and sequencing the whole-genome sequences of E5

| **Primer name** | **Position** | **Sequence (5’-3’)** | **Orientation** | **Source** |
| --- | --- | --- | --- | --- |
| DK001 | 190-565 | CAAGCACTTCTGTTTCCC | Forward | (36) |
| DK004 |  | CACGGACACCCAAAGTAGT | Reverse | (36) |
| E2F | 406-1433 | TGGGACGCTTCAATACTGACA | Forward | This study |
| E2R |  | GCTTCAGCGGTTACTTCGTT | Reverse | This study |
| E3F | 1340-2528 | GTTCCCGAGGCTGAAATG | Forward | This study |
| E3R |  | TTGTTGCGAGTTTGACGG | Reverse | This study |
| EV-BS | 2440-3407 | CTTGTGCTTTGTGTCGGCRTGYAAYGAYTTYTCWG | Forward | (26) |
| EV-BR |  | GGTGCTCACTAGGAGGTCYCTRTTRTARTCYTCCCA | Reverse | (26) |
| E4F1 | 3139-3808 | GAATAAGGAGACCCCAACACC | Forward | This study |
| E4F2 |  | AAAGCGTTTCCAAGTTGTTCG | Reverse | This study |
| E4F | 3398-4493 | AGACACCTAGCCTTACACACT | Forward | This study |
| E4R |  | AATCAAATTGGTTGCCACGGA | Reverse | This study |
| E5F | 4318-5596 | CTCTAACGTGCAGTACTTCGC | Forward | This study |
| E5R |  | AAATTAGTGCCATCCTTGTCC | Reverse | This study |
| E6F | 5408-6457 | GGACCAGCCTTTGAATTCGC | Forward | This study |
| E6R |  | CTAGACTTACCCTTAGCCACC | Reverse | This study |
| E7F | 6212-7418 | GCAACCCTAGACATTAGCACAG | Forward | This study |
| E7R |  | CGAATGCGGAGAATTTACCCCTA | Reverse | This study |
| E8F | 6958-end | CTACCCGCATCCCATCGAC | Forward | This study |
| 7500A |  | GGGGACCACTTTGTACAAGAAAGCTGGG（T）24 | Reverse | (37) |

**TABLE S2** Information on 33 E5 strains’ complete VP1 sequences for genotyping

| **GenBank No.** | **Source** | **Length** | **Isolation country** | **Isolation year** | **Genotype** |
| --- | --- | --- | --- | --- | --- |
| AF083069.1 | GenBank | Full-length | USA | 1954 | A |
| FJ868338.1 | GenBank | VP1 | Australia | 2004 | C1 |
| FJ868339.1 | GenBank | VP1 | Australia | 2005 | B |
| GU142878.1 | GenBank | VP1 | Australia | 1997 | B |
| HG793679.1 | GenBank | VP1 | France | 2012 | C2 |
| HG793680.1 | GenBank | VP1 | France | 2012 | C2 |
| HM775882.1 | GenBank | Full-length | South Korea | 2006 | B |
| JN203687.1 | GenBank | VP1 | India | 2009 | C1 |
| JN203688.1 | GenBank | VP1 | India | 2009 | C1 |
| JN203689.1 | GenBank | VP1 | India | 2009 | C1 |
| JN203690.1 | GenBank | VP1 | India | 2009 | C1 |
| JN203691.1 | GenBank | VP1 | India | 2009 | C1 |
| JX513450.1 | GenBank | VP1 | India | 2009 | C1 |
| JX513451.1 | GenBank | VP1 | India | 2009 | C1 |
| JX513452.1 | GenBank | VP1 | India | 2009 | C1 |
| KF177015.1 | GenBank | VP1 | India | 2009 | C1 |
| LN713449.1 | GenBank | VP1 | Tunisia | 2013 | C2 |
| LN713451.1 | GenBank | VP1 | Tunisia | 2012 | C2 |
| LT883144.1 | GenBank | VP1 | Tunisia | 2012 | C2 |
| MK086217.1 | GenBank | VP1 | France | 2015 | C2 |
| MK086248.1 | GenBank | VP1 | France | 2014 | C2 |
| MK086256.1 | GenBank | VP1 | France | 2014 | C2 |
| MN541010.1 | GenBank | VP1 | China（Jinan-shandong） | 2018 | E |
| MN541031.1 | GenBank | VP1 | China（Jinan-shandong） | 2018 | E |
| MN896924.1 | GenBank | VP1 | USA | 2019 | C2 |
| MN896925.1 | GenBank | VP1 | USA | 2019 | C2 |
| MT641366.1 | GenBank | VP1 | UK | 2017 | D |
| MT950561.1 | GenBank | VP1 | China（Liaocheng-shandong） | 2019 | E |
| MT950565.1 | GenBank | VP1 | China（Liaocheng-shandong） | 2019 | E |
| MT950575.1 | GenBank | VP1 | China（Liaocheng-shandong） | 2019 | E |
| MT950589.1 | GenBank | VP1 | China（Liaocheng-shandong） | 2019 | E |
| SA18-334 | This study | Full-length | China（Luohe-henan） | 2018 | E |
| SA19-378 | This study | Full-length | China（Luohe-henan） | 2019 | E |

**TABLE S3** Nucleotide and amino acid sequence identities with the E5 prototype strain and other E5 strains

| Region | | Homology with the prototype | | | |  | Homology with other E5 strains | | |
| --- | --- | --- | --- | --- | --- | --- | --- | --- | --- |
|  |  | Nucleotide identity (%) | |  | Amino acid identity (%) |  | Nucleotide identity (%) |  | Amino acid identity (%) |
| CDS | | 80.2 | |  | 97.2 |  | 79.6 |  | 96.9 |
| 5’UTR | | 83.8 | |  | - |  | 83.5 |  | - |
| P1 | | 81.5-81.6 | |  | 97.5-97.8 |  | 81.6-81.7 |  | 98.1-98.3 |
|  | VP4 | 84.5 | |  | 100.0 |  | 84.0 |  | 98.5 |
|  | VP2 | 79.6 |  | | 98.0 |  | 81.0 |  | 99.6 |
|  | VP3 | 81.6 |  | | 98.8 |  | 81.8 |  | 99.4 |
|  | VP1 | 81.7 |  | | 95.8 |  | 81.2 |  | 96.2 |
| P2 | | 79.9 |  | | 96.7 |  | 79.1 |  | 95.1 |
|  | 2A | 79.5 |  | | 93.3 |  | 76.8 |  | 93.3 |
|  | 2B | 79.1 |  | | 94.9 |  | 78.1 |  | 97.9 |
|  | 2C | 80.7 |  | | 97.8 |  | 80.1 |  | 96.0 |
| P3 |  | 78.7 |  | | 97.0 |  | 77.6-77.7 |  | 96.8 |
|  | 3A | 78.7 |  | | 98.9 |  | 79.4 |  | 96.9 |
|  | 3B | 78.7 |  | | 100.0 |  | 81.8 |  | 100.0 |
|  | 3C | 79.2 |  | | 97.2 |  | 76.6 |  | 97.8 |
|  | 3D | 78.6 |  | | 96.4 |  | 77.5 |  | 96.2 |
| 3’UTR | | 85.7 |  | | - |  | 80.3 |  | - |

**TABLE S4** The highest similarity of nucleotide sequences of enteroviruses in all sequenced genomic regions of Henan E5 strains using BLAST online

| **Genomic region** | **Type** | **Strain** | **Nucleotide identity（%）** | **Accession number** |
| --- | --- | --- | --- | --- |
| 5’UTR | CVB2 | NS13-004/Nagasaki-JPN/2013 | 93.5 | LC191411.1 |
| VP4 | E5 | E5/SWG63/SD/CHN/2018 | 97.1 | MN541031.1 |
| VP2 | E5 | E5/SWG63/SD/CHN/2018 | 97.2 | MN541031.1 |
| VP3 | E5 | E5/South Korea/2006 | 100.0 | HM775882.1 |
| VP1 | E5 | C48/LC/CHN/2019 | 98.4 | MT950589.1 |
| 2A | E5 | E5/SWG63/SD/CHN/2018 | 97.8 | MN541031.1 |
| 2B | E5 | E5/SWG63/SD/CHN/2018 | 98.7 | MN541031.1 |
| 2C | CVB3 | CVB3/India/2009 | 85.0 | KR107055.1 |
| 3A | E6 | RA/E6/Ahvaz/Iran/2011 | 89.2 | KX619440.1 |
| 3B | E14 | E14/Australia/2012 | 86.4 | MF838734.1 |
| 3C | CVA9 | CVA9/XZ-CHN/2018 | 87.2 | OL519579.1 |
| 3D | CVA9 | CVA9/XZ-CHN/2018 | 100.0 | OL519579.1 |
| 3’UTR | EV-B85 | EV-B85/XJ-CHN/2011 | 95.2 | JX898906.1 |
|  | CVB4 | NA | 95.2 | AF311939.1 |
| P1 | E5 | C24/LC/CHN/2019 | 98.3 | MT950565.1 |
| P2 | E5 | E5/SWG63/SD/CHN/2018 | 96.7 | MN541031.1 |
| P3 | E6 | E6/Australia/2007 | 86.6 | MF678307.1 |


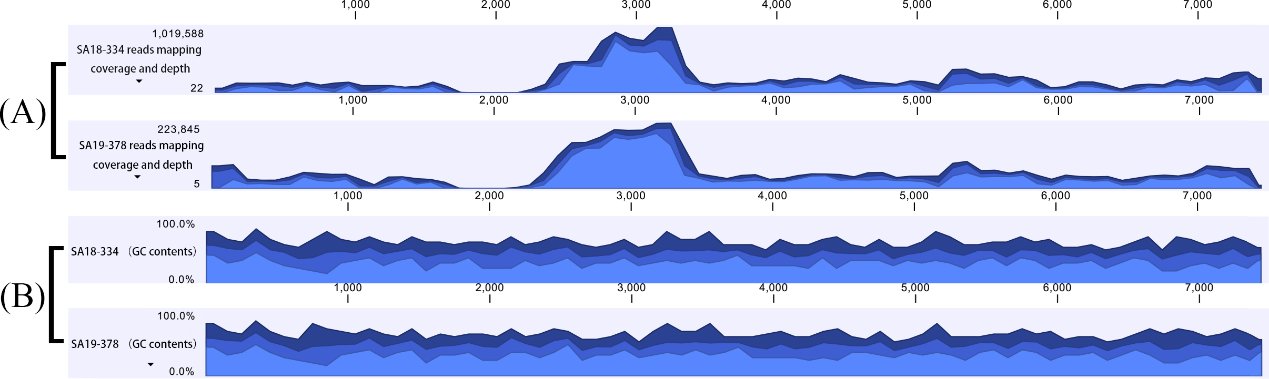


**FIG S1** (A) Mapping of reads against the de novo assembled genomes of the SA18-334 and SA19-378 strains and coverage of reads across the whole genome sequences. (B) SA18-334 and SA19-378 strain GC content graph of the whole genome sequences.
